# QuickStats

**Published:** 2014-07-25

**Authors:** FROM THE NATIONAL CENTER FOR HEALTH STATISTICS

**Figure:**
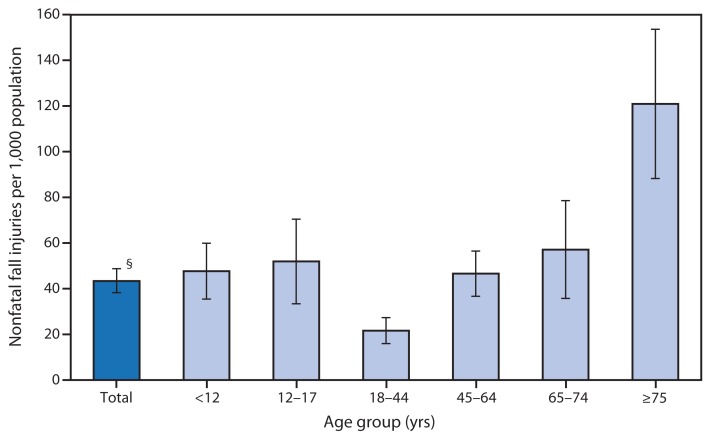
Rate of Nonfatal Fall Injuries Receiving Medical Attention,* by Age Group — National Health Interview Survey,^†^ United States, 2012 * Annualized rate per 1,000 population for fall injury episodes for which a health-care professional was contacted either in person or by telephone for advice or treatment. ^†^ Estimates are based on household interviews of a sample of the noninstitutionalized civilian population. ^§^ 95% confidence interval.

In 2012, the U.S. rate of nonfatal fall injuries receiving medical attention was 43 per 1,000 population. Rates increased with age for adults aged ≥18 years. Adults aged 18–44 years had the lowest rate of falls (22 per 1,000), and the rate for those aged ≥75 years was higher (121 per 1,000) than for all other age groups.

**Source:** Adams PF, Kirzinger WK, Martinez ME. Summary health statistics for the U.S. population: National Health Interview Survey, 2012. Vital Health Stat 2013;10(259).

**Reported by:** Patricia F. Adams, pfa1@cdc.gov, 301-458-4063; Michael E. Martinez, MPH, MHSA; Whitney K. Kirzinger, MPH.

